# Supplementary figures and images for: Antarctic macroalgal-associated amphipod assemblages exhibit long-term resistance to ocean acidification
Source: PeerJ. 2025 May 13;13:e19368. doi: 10.7717/peerj.19368 (PMC12083470; doi:10.7717/peerj.19368)

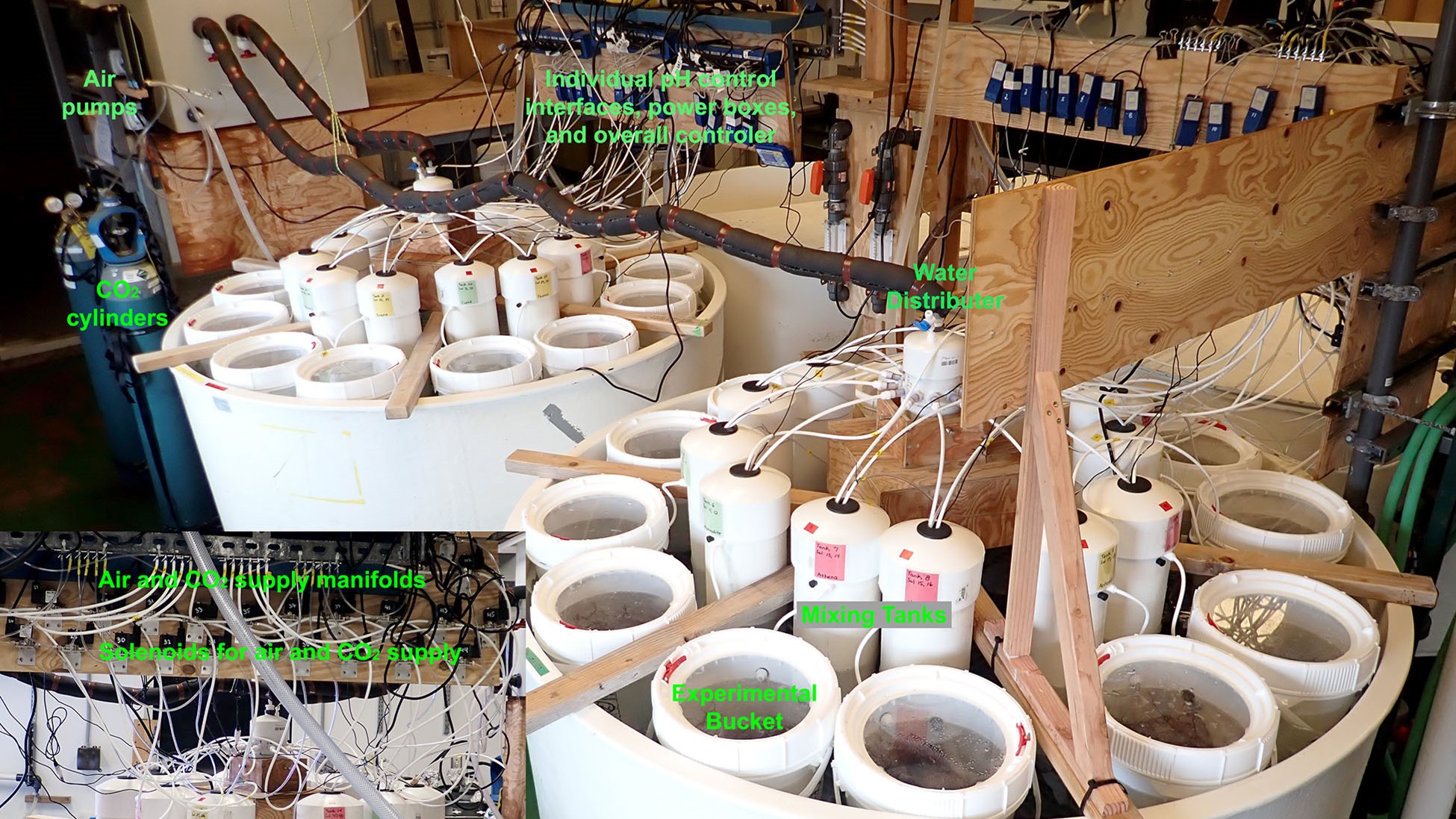

Supplement: Supplemental Information 1 — Twin aquarium tanks each held 24 experimental buckets, 24 mixing tanks, and a water distributor. Water flowed from a header tank into the water distributors which split the water tanks and finally into the experimental buckets before flowing out of the experiment. Each mixing tank contained a pH probe, an air tube, and a tube for a CO2-air mixture which were used to monitor and lower the pH when necessary. This figure was included in Oswalt et al. (2025) as a supplemental figure. [file peerj-13-19368-s001.jpg]
