# Supplementary material for: Antarctic macroalgal-associated amphipod assemblages exhibit long-term resistance to ocean acidification
Source: PeerJ. 2025 May 13;13:e19368. doi: 10.7717/peerj.19368 (PMC12083470; doi:10.7717/peerj.19368)
Supplement: Supplemental Information 2 [file peerj-13-19368-s002.docx]

Table S1. Abundance of amphipods, copepods, ostracods, and isopods in the initial assemblage samples.

| Species/Taxonomic Group | Initial 1 | Initial 2 | Initial 3 | Initial 4 | Initial 5 | Initial 6 | Initial 7 | Initial 8 | AVG |
| --- | --- | --- | --- | --- | --- | --- | --- | --- | --- |
| *Metaleptamphopus pectinatus* | 300 | 328 | 309 | 363 | 176 | 355 | 268 | 388 | 310.86 |
| *Oradarea* spp. | 59 | 59 | 30 | 62 | 25 | 77 | 37 | 80 | 53.63 |
| *Djerboa furcipes* | 15 | 18 | 34 | 15 | 25 | 28 | 25 | 30 | 23.75 |
| *Gondogeneia antarctica* | 29 | 28 | 19 | 23 | 21 | 18 | 22 | 15 | 21.88 |
| *Bovallia gigantea* | 4 | 6 | 5 | 7 | 4 | 9 | 5 | 3 | 5.38 |
| *Prostebbingia gracilis* | 6 | 3 | 5 | 6 | 7 | 6 | 8 | 1 | 5.25 |
| Stenothoidae | 0 | 0 | 1 | 2 | 3 | 10 | 2 | 6 | 3.00 |
| *Jassa* spp. | 1 | 1 | 2 | 0 | 1 | 1 | 0 | 0 | 0.75 |
| *Prothaumatelson nasutum* | 1 | 5 | 0 | 0 | 0 | 0 | 0 | 0 | 0.75 |
| *Paraphimedia integricauda* | 0 | 1 | 0 | 1 | 0 | 1 | 0 | 0 | 0.38 |
| *Gnathiphimedia* sp. | 0 | 0 | 1 | 0 | 0 | 0 | 0 | 0 | 0.13 |
| *Probolisca ovata* | 0 | 1 | 0 | 0 | 0 | 0 | 0 | 0 | 0.13 |
| Lysianasidae | 0 | 0 | 0 | 1 | 0 | 0 | 0 | 0 | 0.13 |
| Unidentifiable Amphipods | 30 | 20 | 23 | 15 | 14 | 15 | 19 | 17 | 19.13 |
| Copepods | 22 | 12 | 10 | 25 | 13 | 16 | 11 | 8 | 14.63 |
| Ostracods | 6 | 8 | 5 | 8 | 3 | 5 | 3 | 3 | 5.13 |
| Isopods | 0 | 0 | 1 | 2 | 0 | 0 | 1 | 2 | 0.75 |
